# Case report: Non-thrombotic iliac vein lesion: an unusual cause of unilateral leg swelling in a patient with endometrial carcinoma

**DOI:** 10.3389/fcvm.2023.1115870

**Published:** 2023-05-02

**Authors:** Jan Zeman, Ritika Kompella, JuYong Lee, Agnes S. Kim

**Affiliations:** ^1^ Department of Internal Medicine, University of Connecticut Health Center, Farmington, CT, United States; ^2^ Calhoun Cardiology Center, University of Connecticut Health Center, Farmington, CT, United States

**Keywords:** peripheral edema, cancer, imaging, vascular disease, intravascular ultrasonography (IVUS)

## Abstract

81-year-old female presented with subacute right lower extremity edema due to iliac vein compression by a markedly enlarged external iliac lymph node later identified as newly relapsed metastatic endometrial carcinoma. The patient underwent a full evaluation of the iliac vein lesion and cancer and had an intravenous stent placed with complete resolution of symptoms post-procedure.

## History of presentation and medical history

An 81-year-old Caucasian female with a history of stage IB high-grade undifferentiated endometrial carcinoma diagnosed 2 years prior and paroxysmal atrial fibrillation (AF) on anticoagulation presented with a two-week history of right leg swelling (Table 1). The edema extended to her knee and was not relieved by conservative measures, including compression stockings. Of note, the patient's endometrial carcinoma was initially treated with hysterectomy, bilateral salpingo-oophorectomy, vaginal brachytherapy, and 6 cycles of chemotherapy with Carboplatin and Paclitaxel. The patient had since been in remission. Her family history was significant for breast cancer and dementia in her mother, atrial fibrillation (AF) and leukemia in her father, and unspecified cancer in her brother. The patient had multiple risk factors for AF—family history of AF, sedentary lifestyle, obesity, hypertension, left atrial enlargement, and previous exposure to carboplatin. She had no significant smoking, alcohol, or illicit drug history. She retired from accounting 20 years ago.

**Table 1:** Timeline of the events.

DATE	EVENT
January 2018	Endometrial biopsy after complaints of severe abdominal pain and vaginal spotting
May 18, 2018	Laparoscopic hysterectomy with bilateral salpingo-oophorectomy for staging purposes after abnormal endometrial biopsy showing endometrial carcinoma
June 15, 2018	Initiation of chemotherapy with Carboplatin and Paclitaxel as well as vaginal brachytherapy for pathologically confirmed stage IB high-grade undifferentiated endometrial carcinoma
November 2018	Completion of chemo-radiation therapy; CT abdomen pelvis showed no evidence of disease
February 2019	Initial diagnosis of atrial fibrillation after an evaluation of sudden onset shortness of breath; started on anticoagulation
June 25, 2020	The patient initially noticed significant right leg swelling
July 8, 2020	Cardiology visit for evaluation of new-onset unilateral leg edema
July 31, 2020	Ultrasound examination of the right lower extremity showed right external iliac vein compression, transthoracic echocardiogram showed normal LVEF, left atrial enlargement, and moderate mitral regurgitation
August 18, 2020	Right iliac vein venogram and right heart catheterization performed
September 2, 2020	Stent was placed in the right iliac vein to relieve the compression
September 21, 2020	Laparoscopy with lymph node biopsy confirmed cancer invasion in the wall of the right iliac vein and the right ureteral wall
April 5, 2023	Ultrasonographic evaluation of the venous stent showed patency.

## Learning objectives

• To broaden the differential diagnosis of unilateral peripheral edema in a patient with a history of cancer.
• To understand the comprehensive evaluation and treatment of NIVL.

## Differential diagnosis

The differential diagnosis of unilateral leg edema includes deep vein thrombosis, cellulitis, Baker's cyst, lower extremity trauma, lymphedema, venous insufficiency, and May-Thurner syndrome.

## Investigations

**Figure 1:**
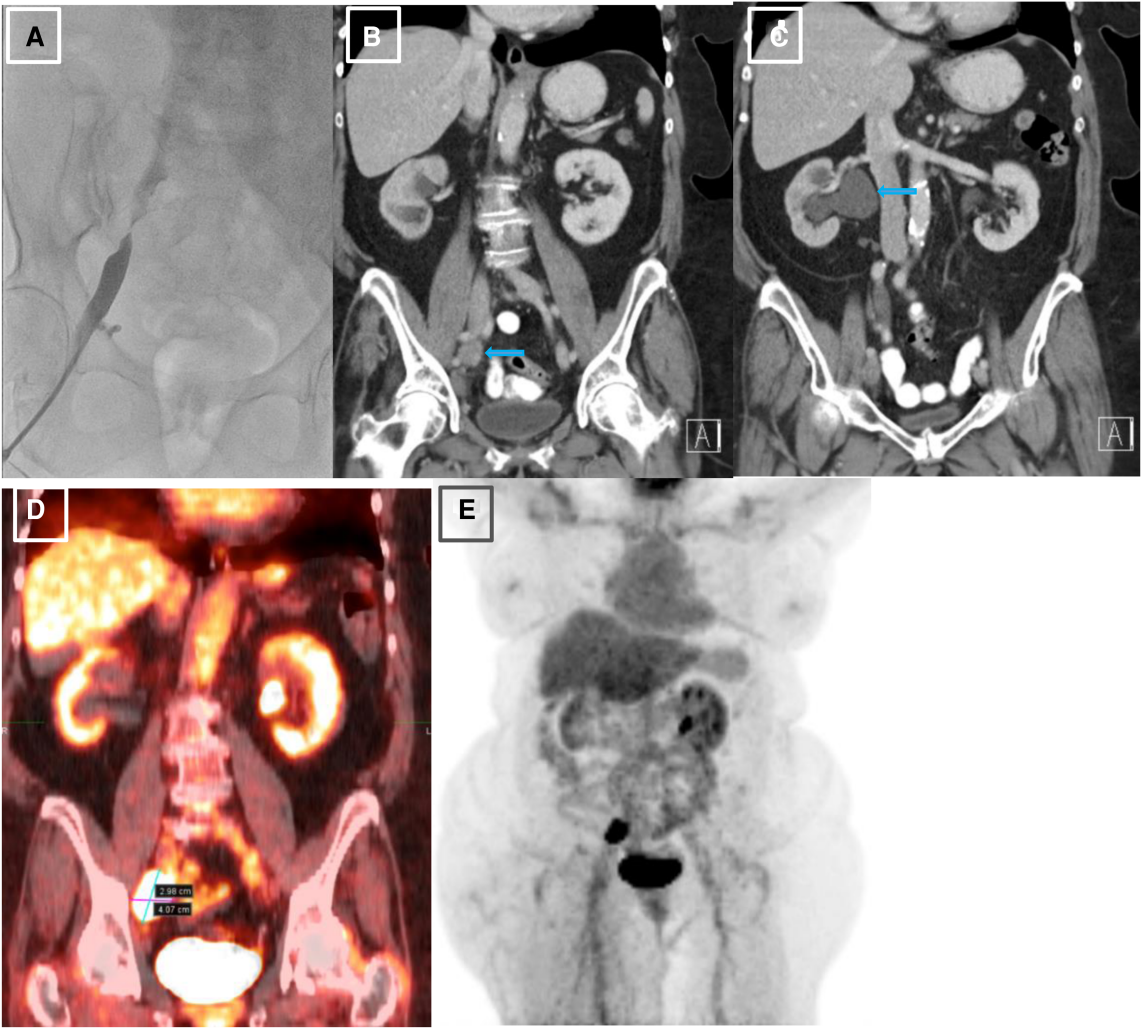
Initial venography, CT and PET imaging of the right external iliac vein. (**A**). Iliac venography demonstrating severe stenosis of right external iliac vein. (**B**). CT abdomen pelvis revealing right external iliac lymphadenopathy (LAD), measuring 2.5 × 3.2 × 2.7 cm, marked by blue arrow. (**C**). CT abdomen pelvis demonstrating severe right sided hydronephrosis due toR ureteral external compression by mass burden, marked by blue arrow. (**D, E**). PET scan redemonstrating R external iliac LAD.

The first step in the patient's diagnostic work-up was a venous Duplex ultrasound test of the right lower extremity, which revealed marked right external iliac vein narrowing with abnormal Doppler waveforms, suggestive of a hemodynamically significant obstructive lesion secondary to external compression. A transthoracic echocardiogram revealed normal left ventricular size and systolic function, mild left atrial dilation, grade 1 diastolic dysfunction, moderate mitral regurgitation, and an elevated estimated right atrial pressure based on the dilated inferior vena cava. To measure the intracardiac pressures, rule out pulmonary hypertension, and better characterize the iliac vein stenosis, the patient underwent right heart catheterization (RHC) with iliac venography. The iliac venogram revealed severe stenosis of the right external iliac vein (Figure 1A), while RHC showed normal intracardiac pressures (normal RAP, mean PAP, and PCWP).

Given her history of endometrial carcinoma, the possibility of extrinsic compression of the iliac vein by tumor was considered. Thus, a computed tomography (CT) scan of the abdomen/pelvis with and without intravenous (IV) contrast was obtained. The results indicated a markedly enlarged and necrotic right external iliac lymph node (2.5 × 3.2 × 2.7 cm) which was causing external compression of, and likely invading, the right iliac vein (Figure 1B). Significant compression of the right ureter associated with severe right-sided hydronephrosis (Figure 1C) and right pelvic sidewall lymphadenopathy were seen as well. Next, a full-body PET scan was ordered for staging purposes, demonstrating metabolic activity in the right external iliac lymph node and an area of increased activity in the left adrenal gland (Figures 1D, E). After a multidisciplinary discussion, the patient was offered an endovascular intervention of the iliac vein with stenting to improve her refractory right lower extremity edema.

## Management

**Figure 2:**
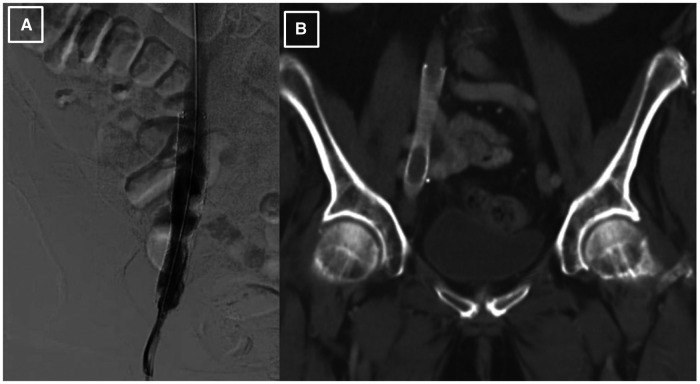
Post-procedural imaging of the stented right external iliac vein. (**A**). Iliac venography status post IVUS-guided visualization and right external iliac vein balloon-angioplasty with successful placement of VICI 14 × 90 mm stent and resultant patency. (**B**). CT abdomen pelvis demonstrating presence of stent within the R external iliac vein prior to abdominal debulking procedure.

Since there was a high probability of cancer invading the vessel wall and the right ureter, a decision was made for palliative endovascular stent placement to provide symptomatic relief and to avoid the risks of an open surgical approach, including intra-operative and post-operative bleeding. The patient was taken to the cardiac catheterization laboratory, where an intravascular ultrasound (IVUS) was performed to visualize the lesion and to measure the lesion length and the reference diameter for choosing the correct size of the stent. After the lesion was crossed with a 0.035 angled glide guide wire, the patient underwent right external iliac vein balloon angioplasty followed by the successful placement of a VICI 14 × 90 mm stent, VENITI, INC., Fremont, CA. Post-dilation was done with a 10 × 60 mm balloon at 8 atmospheres and a 14 × 60 mm balloon at 4 atmospheres. The follow-up IVUS showed a re-canalized iliac vein with normal venous flow and 100 mm^2^ of luminal gain at the previously occluded point. The re-canalization was additionally re-demonstrated on an abdominal/pelvic CT scan (Figures 2A, B).

## Follow-up

The patient's right lower extremity edema completely resolved after the intervention. The patient was started on aspirin 81 mg daily for three months to prevent stent thrombosis and allow its endothelization. A few weeks later, the patient underwent laparoscopy with retroperitoneal lymph node biopsy and tumor debulking. The surgeon released the ureteral compression but was unable to remove the obstructing lymph node, as it was invading the iliac vein wall with a high risk of bleeding. The patient was followed by interventional cardiology every six months with Doppler imaging, with vein patency noted during two years of follow-up.

## Discussion

The development of lower extremity edema, as other types of edema, occurs by three main mechanisms, all of which are caused by fluid shifts from the intravascular to the interstitial space: an increase in intravascular hydrostatic pressure, a decrease in hydrostatic oncotic pressure or increased capillary permeability. The diagnostic work-up for leg swelling depends on the time of onset and its laterality. As seen in this patient, the development of an acute or subacute unilateral lower extremity edema can occur over the span of hours or weeks, and the differential diagnosis includes deep vein thrombosis (DVT), musculoskeletal injury, ruptured popliteal cyst, or venous compression (1). Patients with cancer have a high risk of developing venous thromboembolism (VTE) for several reasons: cancer is associated with high a pro-inflammatory and hypercoagulable state, tumor mass can cause venous external compression, and certain anticancer drugs can increase the risk of VTE. Anticancer drugs that have been reported to increase VTE risk include Thalidomide, Lenalidomide, and Bevacizumab (2). These medications are linked to an increased risk of VTE including DVT and arterial events (2). Thalidomide, particularly when used with dexamethasone, has been associated with up to 28% DVT rates, with additional risk factors including combined use with Doxorubicin, newly diagnosed disease, and Chromosome 11 abnormalities (2). Lenalidomide may have VTE rates as high as 75% (2). Bevacizumab, an anti-angiogenic, has been linked to an increased risk of arterial and venous events (2). Platinum-based compounds, such as cisplatin and carboplatin, can also cause arterial and venous thromboembolism (3). In patients with unprovoked DVT, age-appropriate cancer screening should follow the treatment of DVT. In the case of bilateral lower extremity edema in a patient with cancer, the differential diagnosis should also include thrombosis within, or extrinsic compression of, the inferior vena cava.

Iliac vein compression syndrome, a subtype of NIVL, also known as May-Thurner syndrome (MTS) is a condition mostly seen in young women who present with left leg swelling due to the compression of the left common iliac vein by the right common iliac artery at the level of the lumbar spine. The prevalence of MTS is estimated to be between 15%–30% based on autopsy data. The vast majority of these patients remain asymptomatic with only 2%–5% of patients developing a DVT. Long-standing MTS often results in chronic venous disease (4). In contrast to MTS, NIVL can occur in any segment of the iliac vein caused by other etiologies such as extrinsic compression by tumor, mass, spine spurs, or calcified atherosclerotic iliac arteries (5).

The first-line imaging modality used in diagnosing NIVL or evaluating unilateral leg swelling in patients with cancer is Doppler ultrasound of the iliac and other lower extremity veins. The test is noninvasive, easily accessible, and readily available. However, its diagnostic value may be limited in lesions with lower severity or in patients with obesity due to technical limitations. For typical MTS, CT pelvis with IV contrast is not sensitive enough to diagnose the compressed iliac vein due to limited visibility, but it is a good modality for ruling out extrinsic compression as in NIVL. CT venography with contrast is useful to further differentiate thrombotic from non-thrombotic intravenous lesions (6). Invasive venography is a useful diagnostic tool for identifying occlusion or severe stenosis of iliac veins, as demonstrated in our case. However, its effectiveness may be limited in detecting luminal narrowing, resulting in potentially missed diagnoses of NIVL due to its low sensitivity in this regard. Specifically, the diagnostic quality may be compromised in cases of non-occlusive or partially narrowed iliac vein lesions. Therefore, in instances where clinical indications strongly suggest NIVL, such as in our case, the use of intravascular ultrasound (IVUS) is warranted for a more accurate diagnosis as IVUS can provide a 360^o^ view within the vessel itself, which provides greater anatomic detail of the affected region (7). In fact, it has been demonstrated to approximate the morphology of the lesion, affording greater predictive value for stent sizing compared with the CT venogram alone (8, 9). IVUS was found to have a diagnostic sensitivity of > 90% for lesion detection when compared with CT venography, which has a 68% sensitivity (6).

The spectrum of treatments available for NIVL largely depends on the severity and persistence of the patient's symptoms. Conservative management includes compression stockings with leg elevation and calf muscle exercises. In refractory cases, endovascular repair with stent placement is pursued (10). Many studies have demonstrated an overall improvement in the quality of life and reduction in symptom severity with the use of venous stenting, such that it is nearly considered to be the gold standard for patients with high symptom burden (5, 9, 11).

A specific subgroup of NIVL called cancer-associated vein obstruction (CAVO) has recently garnered attention (12). A few studies have shown that percutaneous intervention is effective in reducing symptoms of swelling and discomfort with a low risk of complications (13). One group studied the efficacy of treating CAVO with an endovascular venous stent combined with linear Iodine-125 radioactive seeds strand. The new technique yielded longer patency of the stent and lower symptom burden without affecting survival (14).

## Conclusions

In patients with cancer, DVT is the most likely cause of acute or subacute unilateral leg edema and should be ruled out first before considering other less common etiologies, such as NIVL. The latter condition may not be widely recognized by general providers. The recommended diagnostic workup consists of a duplex ultrasound of the deep iliac venous system, which often demonstrates abnormal venous Doppler waveforms, and a CT venogram of the pelvis to potentially visualize the iliac vein compression. It is important to remember that a negative CT venogram does not rule out NIVL due to its low diagnostic sensitivity. When there is a high index of suspicion for NIVL, invasive iliac venography with IVUS evaluation is instrumental in leading to the correct diagnosis and offering the option of therapeutic endovascular venous stenting. After multidisciplinary discussion and shared decision-making with the patient, endovascular intervention vs. surgical approach vs. medical management is chosen depending on the clinical situation.

## Data Availability

The original contributions presented in the study are included in the article, further inquiries can be directed to the corresponding author.
